# Bacterial Lipoteichoic Acid Attenuates Toll-Like Receptor Dependent Dendritic Cells Activation and Inflammatory Response

**DOI:** 10.3390/pathogens9100825

**Published:** 2020-10-08

**Authors:** Suguru Saito, Alato Okuno, Duo-Yao Cao, Zhenzi Peng, Hui-Ya Wu, Shu-Hui Lin

**Affiliations:** 1 Bio-fluid Biomarker Center, Graduate School of Medical and Dental Sciences, Niigata University, Niigata 9502181, Japan; 2 Department of Biomedical Sciences, Cedars-Sinai Medical Center, Los Angeles, CA 90048, USA; zzpivy@csu.edu.cn; 3 College of Animal Science and Technology, Northwest A&F University, Shaanxi 712100, China; 9527caojunn@gmail.com; 4 Department of Health and Nutrition, Tsukuba international university, Ibaraki 3000051, Japan; a-okuno@tius.ac.jp; 5 Department of Food and Life Sciences. College of Agriculture, Ibaraki University, Ibaraki 3000393, Japan; 6 Institute of Medical Sciences, Xiangya Hospital, Central South University, Changsha 410008, China; 7 College of Health Science, Trans World University, Douliu 64063, Taiwan; Wuhuiya0815@gmail.com; 8 Medical Science International, Taipei 115, Taiwan; shjlin.usm.med@gmail.com

**Keywords:** lipoteichoic acid (LTA), *Staphylococcus aureus* (*S. aureus*), toll-like receptor (TLRs), dendritic cell (DCs), immunosuppressive, effector CD4+ T cells, acute skin inflammation

## Abstract

Toll-like receptor (TLR) signaling is an indispensable factor in immune cells activation. Many TLR ligands have been identified, and were characterized the immunological functions such as inflammatory cytokine production in immune cells. However, the anti-inflammatory response in TLR ligand-mediated manner is poorly understood. In this report, we show that bacterial lipoteichoic acid (LTA), which is a TLR2 ligand from gram-positive bacteria including *Staphylococcus aureus* (*S. aureus*), suppresses TLR-mediated inflammatory response in dendritic cells (DCs). The TLR ligand-induced Tumor Necrosis Factor-alpha (TNF-α) production was suppressed in the bone marrow derived dendritic cells (BMDCs) by co-treatment of LTA. The cellular activation, which was characterized as upregulations of CD80, CD86 and major histocompatibility complex II (MHC II) expression, was also suppressed in the TLR ligand stimulated BMDCs in the presence of LTA. While LTA itself didn’t induced both TNF-α production and upregulation of cell surface markers. The LTA mediated immunosuppressive function was abolished by TLR2 blocking in lipopolysaccharide (LPS)-stimulated BMDCs. Furthermore, LTA also showed the immunosuppressive function in the generation of IFN-γ+CD4+ T (Th1) cells by attenuation of antigen presenting activity in the BMDCs. In the imiquimod (IMQ)-induced acute skin inflammation, LTA suppressed the inflammation by downregulation of the activation in skin accumulated DCs. Thus, LTA is a TLR2 dependent immunological suppressor against inflammatory response induced by other TLR ligands in the DCs.

## 1. Introduction

Toll-like receptor (TLR) is a pattern recognition receptor for various ligands, and it is widely expressed in immune cells [1]. The ligand stimulation for each TLR induces activation in the immune cells which is characterized by upregulation of cytokine production, surface marker expression and cell proliferation [2,3]. TLR signaling is an indispensable factor to induce innate immune response against pathogen to prevent serious infectious disease [4]. Dendritic cell (DC), one of the myeloid origin immune cells, expresses several types of TLRs and these receptors are working for the regulation of DCs activity [5]. As a professional antigen presenting cells (APCs), DCs are activated by antigenic substances which captured by both phagocytotic and pinocytotic manner, then the cells present the processed antigen into T cells to activate adaptive immune response against the target with high specificity [6]. DCs activation status can be augmented by TLR stimulation so that the expression of the molecules directory related with antigen presentation, such as major histocompatibility complex class II (MHCII), CD80 and CD86, are upregulated to promote antigen presentation in activation of T cells [7,8]. The TLR signaling is generally thought as an unidirectional event which means the signal works only for activation in the cells. However, other suppressive mechanism via TLR with specific ligand has gradually been revealed recently. Lipoteichoic acid (LTA) from gram-positive bacteria recognized by TLR2, however, the recognition itself didn’t induce inflammatory response in the keratinocytes [9]. On the contrary, the LTA signal suppressed another TLR signaling, such as TLR3 by poly (I:C) stimulation which characterized by suppression of inflammatory cytokine production in the cells [9]. It was an interesting finding of LTA’s anti-inflammatory function in TLR-mediated manner, however, no other clear evidence hasn’t been reported in the immune cells yet. In addition, it has been expected to confirm that the efficiency of LTA’s immunosuppressive effect in other TLR ligands mediated inflammation.

In this report, we show that LTA, originated from *Staphylococcus aureus* (*S. aureus*), suppressed TLR signal dependent inflammatory response in the DCs. The inflammatory cytokine production was dramatically suppressed by LTA treatment in TLR ligand stimulated murine bone marrow derived dendritic cells (BMDCs). In this response, CD80, CD86 and MHC class II expressions were all downregulated by LTA treatment in the cells. LTA treatment itself was no-inflammatory event confirmed as fewer cytokine production, which was same level as vehicle control, than other ligands stimulation in the BMDCs. The LTA based suppressive effect against lipopolysaccharide (LPS)-induced cytokine production and surface molecule upregulation were TLR2 dependent manner which confirmed by in vitro TLR2 blocking assay. The antigen specific IFN-γ+CD4+ T (Th1) cells generation was suppressed by LTA treatment in BMDCs. Imiquimod (IMQ)-induced acute skin inflammation was suppressed by co-treatment of LTA on the mice ears. In this suppressive effect, TNF-α production was decrease in the skin accumulated DCs.

These findings provide a novel understanding of immunosuppressive effect by LTA against other TLRs-induced inflammation in the immune cells such as DCs.

## 2. Results

### 2.1. LTA Suppressed TLR-Mediated Cytokine Production in BMDCs

**Figure 1:**
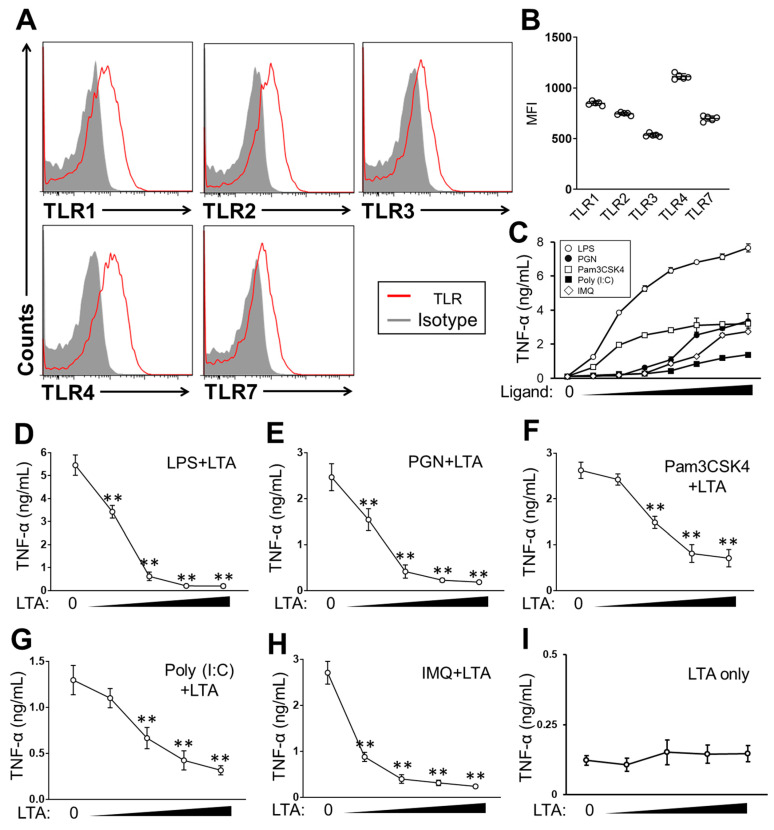
LTA suppresses TLR-dependent cytokine production in (BMDCs). (**A**, **B**) The characterization of TLR expression in BMDCs. TLR1, 2, 3, 4 and 7 were analyzed by flow cytometry. The representative image (**A**) and the mean fluorescence intensity (MFI) (**B**) of each TLR expression in the analysis. (**C**) BMDCs were stimulated with TLR ligands at 37 °C for 24 h, then the TNF-α production was measured by ELISA. Ligand concentration was 0.01, 0.05, 0.1, 0.5, 1, 5 and 10 ug/mL, respectively. (**D**–**I**) BMDCs were stimulated with TLR ligand in the presence or absence of LTA (**D**–**H**). Some cells were treated with LTA-only (**I**). The concentration of TLR ligand was LPS: 100 ng/mL, PGN: 10 ug/mL, Pam3CSK4: 100 ng/mL, Poly (I:C): 10 ug/mL or IMQ: 10 ug/mL, respectively. LTA concentration was 0.1, 0.5, 1 and 5 ug/mL at each point. The culture was incubated at 37 °C for 24 h, then, the TNF-α production was measured by ELISA. Data are shown as the mean ± SEM of at least five samples in independent experiments. Student’s *t*-test was used to analyze data for significant differences. Values of ** *p* < 0.001 were regarded as significant.

We firstly characterized TLR expression in murine BMDCs, which were prepared from C57/BL6J mice BM, used in this study. The BMDCs expressed TLR1, 2 and 4 on the surface, as well as TLR3 and 7 in the intracellular (Figure 1A). The expression level was different in each TLR, and TLR4 expression was the most obvious, followed by TLR1, 2 and 7 in the cells (Figure 1B). The stimulation of the BMDCs with ligands, such as Pam3CSK4 (TLR1/2), Peptidoglycan (PGN) (TLR2/4), LPS (TLR4), Poly (I:C) (TLR3) and IMQ (TLR7), all induced TNF-α production in a clearly dose-dependent manner in the cells (Figure 1C). The argument for cytokine production was correlated with the level of TLR expression, which means that TLR4 stimulation induced the most abundant TNF-α production in the BMDCs. These results showed that extracellular or intracellular TLRs were stimulated by each specific ligand respectively in the BMDCs. We found a strong suppressive effect of LTA in TLR-dependent cytokine production in the BMDCs. BMDCs treated with TLR ligands combined with LTA showed significant suppression of TNF-α production (Figure 1D–H). The TNF-α production induced with LPS, PGN or IMQ was all dramatically suppressed by co-treatment of LTA (Figure 1D,E,H). Although the suppression was not as dramatic as other types of TLR ligand stimulation, the TNF-α production was also suppressed by co-treatment of LTA in Pam3CSK4 or Poly (I:C)-stimulated BMDCs, respectively (Figure 1F,G). The LTA-dependent suppressive effect in TNF-α production was clearly dose-dependent in the BMDCs. The most effective range of LTA concentration was slightly different in each type of TLR ligand stimulation; however, even a low concentration of LTA (100 ng/mL) showed a strong suppressive effect in the TLR-dependent cytokine production in the BMDCs. Notably, LTA itself hardly induced TNF-α production in the BMDCs throughout the dose used in this study, and the TNF-αproduction occurred at an almost identical level to the vehicle control (Figure 1I).

Taken together, this shows that LTA suppresses TLR-dependent cytokine production in DCs.

### 2.2. The Immunosuppressive Effect of LTA Is TLR2-Dependent in BMDCs

**Figure 2:**
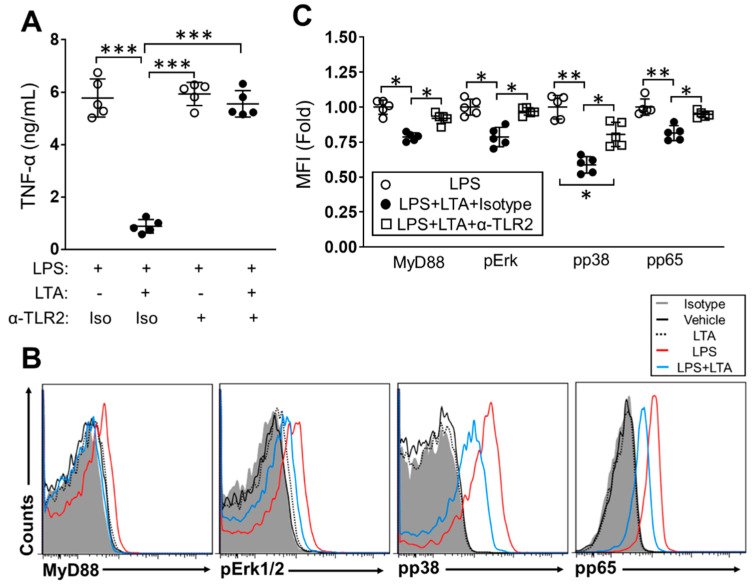
The immunosuppressive effect of LTA is TLR2-dependent in LPS-stimulated BMDCs. (**A**–**C**) BMDCs were pre-treated with anti-TLR2 mAb or isotype antibody at 37 °C for 1 h and then stimulated with LPS (100 ng/mL) combined with LTA (5 ug/mL) or vehicle control. The culture was further incubated at 37 °C for 24 h, then the TNF-α production was measured by ELISA (**A**). The expression of MyD88, pErk1/2, pp38 and pp65 was analyzed by flow cytometry (**B**, **C**). Data are shown as the representative and mean ± SEM of five samples in independent experiments. Student’s *t*-test was used to analyze data for significant differences. Values of * *p* < 0.05, ** *p* < 0.01 and *** *p* < 0.001 were regarded as significant.

We investigated the responsive receptor and signaling pathway in the LTA-mediated anti-inflammatory effect of BMDCs. Since LTA has been characterized as a TLR2 ligand [10], we performed TLR2 blocking in BMDC stimulation by using anti-TLR2 mAb. The blocking efficiency of anti-TLR2 mAb was investigated in the cytokine production of TLR ligand-stimulated BMDCs. PGN, a TLR2 ligand, induced TNF-α production in the culture treated with isotype antibody or vehicle control. On the other hand, cytokine production was suppressed by TLR2 blocking in PGN-stimulated cells. The anti-TLR2 mAb treatment did not affect cytokine production in LPS-stimulated cells. LTA hardly induced TNF-α production, so no obvious inhibitory influence was observed in the LTA-treated cells with anti-TLR2 mAb (Supplementary Figure S1). In the LPS-stimulated BMDCs, LTA dramatically suppressed TNF-α production in the presence of the isotype antibody. On the other hand, TLR2 blocking abolished the suppressive effect of LTA in the LPS-induced TNF-α production of BMDCs (Figure 2A). The suppressive effect of LTA was also abolished by TLR2 blocking in the BMDCs stimulated with another TLR ligand, such as IMQ, so the TNF-αproduction was recovered in the cells as compared to isotype antibody treatment (Supplementary Figure S2).

We next investigated the signaling pathway which was directly affected by the inhibitory effect of LTA in the LPS-stimulated BMDCs. The LPS-dependent TLR4 signaling activates downstream factors such as myeloid differentiation primary response 88 (MyD88), extracellular signal-regulated kinase (Erk) and nuclear factor kappa-light-chain-enhancer of activated B cells (NF-kB) to induce inflammatory cytokine production [11]. In checking the blocking effect of anti-TLR2 mAb, TLR2 inhibition clearly suppressed PGN-induced upregulation of MyD88, phospho-Erk1/2 (pErk1/2), phospho-p38 (pp38) and phospho-p65 (pp65) in the BMDCs, while the blocking of TLR2 had no influence in the LPS stimulation. In addition, the signal transducing proteins hardly responded to the LTA treatment, so the blocking of TLR2 did not show any downregulation of these factors in the cells (Supplementary Figure S3). LPS stimulation upregulated MyD88, pErk1/2, pp38 and pp65 expression in BMDCs (Figure 2B,C). The expression of each protein was downregulated in the presence of LTA in LPS-stimulated BMDCs, respectively (Figure 2B,C). TLR2 blocking abolished the suppressive effect of LTA, so LPS-induced upregulation of MyD88, pErk1/2, pp38 and pp65 were all sustained in the BMDCs as same level as in the cells treated with LPS-only (Figure 2C).

Thus, the suppressive effect of LTA in the inflammatory response is a TLR2-dominant mechanism in TLR ligand-stimulated DCs.

### 2.3. LTA Suppresses TLR-Dependent Cellular Activation in BMDCs

**Figure 3:**
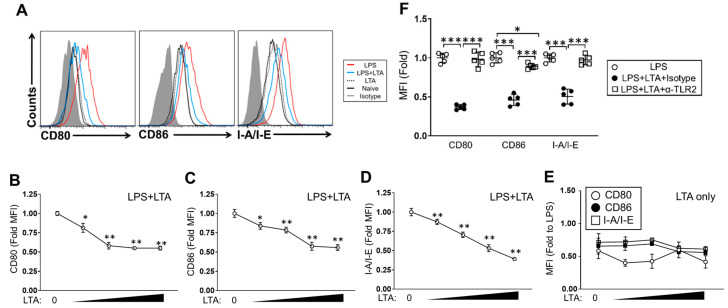
LTA suppresses LPS-induced surface molecule upregulation in BMDCs. (**A**–**E**) BMDC cells were stimulated with LPS (100 ng/mL) in the presence or absence of LTA (**A**–**C**, **E**) or LTA only (**D**, **E**). LTA concentration was 0.1, 0.5, 1 and 5 ug/mL at each point. The culture was incubated at 37 °C for 24 h, then CD80, CD86 and I-A/-E expression on the cell surface was analyzed by flow cytometry. (**F**) BMDCs were pre-treated with anti-TLR2 mAb or isotype antibody at 37 °C for 1 h and then stimulated with LPS (100 ng/mL) combined with LTA (5 ug/mL) or vehicle control. The culture was further incubated at 37 °C for 24 h, then CD80, CD86 and I-A/I-E expression on the cell surface was analyzed by flow cytometry. Data are shown as the representative and mean ± SEM of five samples in independent experiments. Student’s *t*-test was used to analyze data for significant differences. Values of * *p* < 0.05, ** *p* < 0.01 and *** *p* < 0.001 were regarded as significant.

As a professional antigen-presenting cells, a DCs express antigen-presenting molecules and co-stimulatory molecules such as MHC class II, CD80 and CD86 [12]. These expressions are upregulated by antigen simulation as well as TLR ligand stimulation [13]. The BMDCs stimulated with LPS showed upregulation of CD80, CD86 and I-A/I-E as compared to vehicle control treatment (Figure 3A). The LPS-induced upregulation of these expressions were significantly suppressed by LTA in a dose-dependent manner (Figure 3A–D). On the other hand, LTA treatment did not induce any upregulation of surface molecules at all in the dose range used in this experiment, and the expression levels of molecules were obviously lower than those of LPS-stimulated cells (Figure 3E). To investigate the responsive pathway in LTA-mediated downregulation of surface molecules in TLR ligand-stimulated BMDCs, we again performed a TLR2 blocking assay. In the efficiency check of TLR2 blocking, PGN-induced upregulation of CD80, CD86 and I-A/I-E was suppressed in the BMDCs treated with anti-TLR2 mAb. LPS-induced these surface molecule upregulations were all sustained, even in the presence of anti-TLR2 mAb. As same as the case for cytokine production and signaling pathway activation analysis, LTA treatment hardly induced CD80, CD86 and I-A/I-E upregulation in the BMDCs, so we could not confirm any downregulation of this expression in the cells treated with anti-TLRs mAb (Supplementary Figure S4). The LTA-mediated downregulation of CD80, CD86 and I-A/I-E was abolished in the LPS-stimulated BMDCs with TLR2 blocking. All surface molecule expressions were sustained at the same level as LPS-only treatment (Figure 3F). The downregulation of surface molecule expression by LTA treatment was also confirmed in IMQ-stimulated BMDCs (Supplementary Figure S5).

Thus, LTA suppresses cellular activation not only in the cytokine production but also in the surface molecule expression, which are indispensable factors in immunological functions of DCs, such as antigen presentation.

### 2.4. LTA Suppresses Antigen-Specific Effector CD4+ T Cells Generation

**Figure 4:**
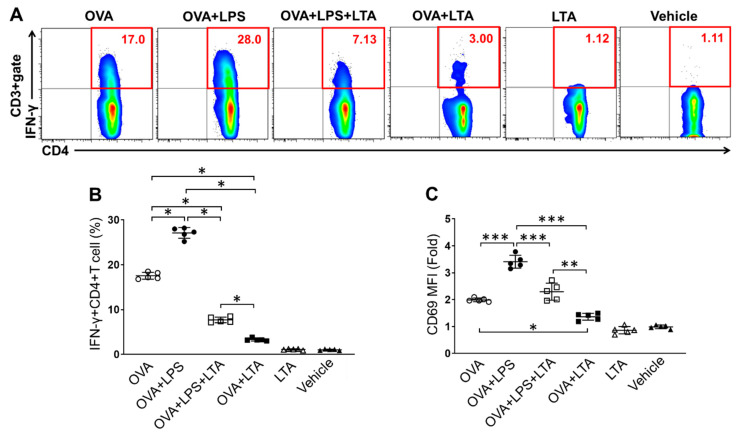
LTA suppresses antigen-specific effector CD4+ T cell generation. (**A**–**C**) BMDCs were cocultured with splenic CD4+ T cells isolated from OT-II mice in the presence of OVA peptide (OVA_323–339_: 10 ug/mL). Some cultures were further treated with LPS (100 ng/mL), LPS+LTA (5 μg/mL) or LTA. The samples treated with LTA only or vehicle were negative control (no-Ag). The cultures were incubated at 37 °C for 72 h. At the last 5 h, the cells were re-stimulated with Phorbol 12-myristate 13-acetate (PMA)/ionomycin, then IFN-γ+CD4+ T (Th1) cells (**A**, **B**) and CD69 expression on the population (**C**) were analyzed by flow cytometry. Data are shown as the representative and mean ± SEM of five samples in independent experiments. Student’s *t*-test was used to analyze data for significant differences. Values of * *p* < 0.05, ** *p* < 0.01 and *** *p* < 0.001 were regarded as significant.

To investigate the influence of LTA-mediated suppression of DCs activation in effector T cells generation, we performed an in vitro antigen presentation assay. The IFN-γ+CD4+ T (Th1) cell population generated by BMDCs in an antigen-specific manner was analyzed by flow cytometry (Figure 4A,B). BMDCs generated Th1 cells in the presence of ovalbumin (OVA) peptide in the culture. The Th1 cell population was increased in the culture treated with LPS, and the percentage was significantly increased as compared to that of OVA peptide-only. Interestingly, the Th1 cell population was decreased in the culture co-treated with LPS and LTA. The culture treated with LTA also showed the suppression of Th1 cells generation. These samples both showed significant decreasing in the percentage of Th1 cells as compared to the culture with LPS-only and even OVA peptide-only. The culture treated with LTA-only did not show Th1 cells generation and the percentage was similar to that of the vehicle control. The suppressive effect of LTA was confirmed in IMQ-stimulated BMDCs in the generation of Th1 cells. The antigen-specific Th1 cells generation was enhanced in IMQ-stimulated BMDCs, however, it was significantly suppressed by co-treatment of LTA in the culture (Supplementary Figure S6). The cellular activation was investigated by analysis of CD69 expression in the Th1 cells. The expression level was completely corelated with the level of Th1 cells generation in each culture. The CD69 expression was upregulated in the culture with LPS treatment, while the level was significantly downregulated in the presence of LTA (Figure 4C). These results might originate from the downregulation of antigen-presenting molecules by LTA treatment in the BMDCs (Figure 3).

Thus, LTA suppresses antigen-specific Th1 cells generation by the downregulation of antigen-presenting molecules in DCs.

### 2.5. LTA Attenuates TLR Ligand-Induced Acute Skin Inflammation with Suppression of Inflammatory Response in DCs

**Figure 5:**
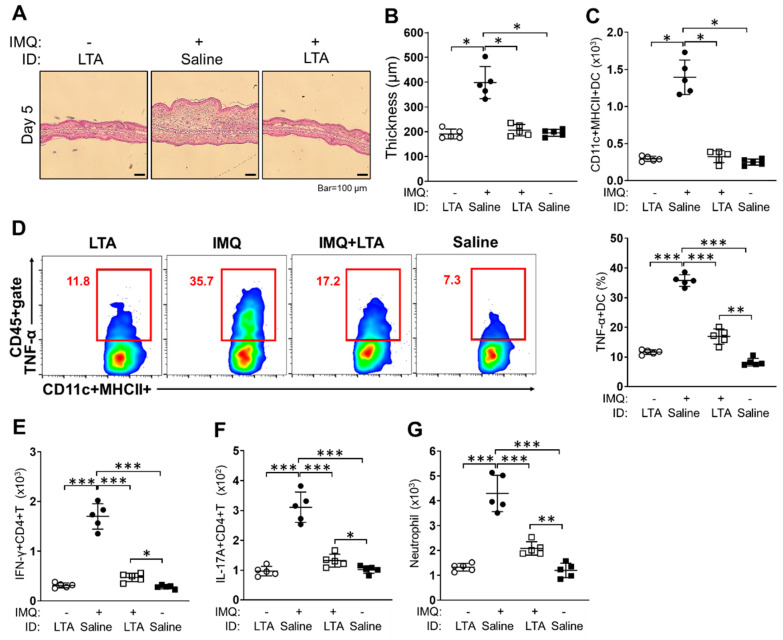
IMQ-induced acute skin inflammation is suppressed by co-treatment of LTA. The mice were treated with 5% IMQ cream or vehicle cream on the ears for 5 days. The mice received intradermal (ID) injection of LTA or saline into the ears in the meantime. (**A**, **B**) Ear thickness at day 5 of the treatment. The ears were corrected and stained with H-E (**A**). The thickness was measured under microscope (**B**). Bar = 100 μm. (**C**–**G**) The characterization of skin-accumulated immune cells in IMQ-induced acute skin inflammation. The leukocytes were isolated from the treated ears, then the sample was analyzed by flow cytometry. The number of CD11c+MHC class II+ DCs in CD45+gate **(C)**, the percentage of TNF-α producing DCs (**D**), the number of IFN-γ+CD4+ T cells (**E**) and IL-17A+CD4+ T cells (**F**) in CD45+CD3+gate, the number of neutrophils (Ly-6G+Ly-6C+/dim) in CD45+CD11b+gate (**G**) were calculated in the analysis, respectively. Data are shown as the representative and mean ± SEM of five samples in independent experiments. Student’s *t*-test was used to analyze data for significant differences. Values of *p < 0.05, ** *p* < 0.01 and *** *p* < 0.001 were regarded as significant.

To investigate the suppressive effect of LTA in tissue inflammation, we established an acute skin inflammation model by using a TLR ligand. The mice ears treated with IMQ showed severe skin inflammation. The IMQ-treated ears had obvious immune cells accumulation in dermal layers in histology, and the thickness was significantly increased in the ears as compared to control treatment (saline) (Figure 5A(middle),B). On the other hand, the inflammation and thickness were both attenuated in the ears received a combined treatment of IMQ and LTA. The thickness was markedly decreased in the ears as compared to that of IMQ-only treatment (Figure 5A(right),B). LTA-only treatment did not show any inflammation in histology, and the ear thickness had no difference compared with control treatment (Figure 5A(left),B). The acute skin inflammation induced by other TLR ligand treatments were also suppressed by co-treatment with LTA. The ear thickness induced by LPS, PGN, Pam3CSK4 and Poly (I:C) treatment were all significantly decreased by co-treatment of LTA (Supplementary Figure S7). The accumulation of DCs was promoted in the IMQ-treated ears. The number of DCs was significantly increased in the IMQ-treated ears as compared to that of the control treatment. On the other hand, the DC accumulation was suppressed by LTA in the IMQ-treated ears, and the skin-accumulated DCs were reduced to the same number as the control-treated ears (Figure 5C). The inflammatory response in the tissue-accumulated DCs, such as TNF-α production, was promoted in IMQ treated ears, however, it was suppressed by co-treatment of LTA in the ears. The percentage of TNF-α producing DCs was significantly increased in IMQ-treated ears as compared to that of the LTA-only and control treatments. The co-treatment of IMQ and LTA suppressed the increasing of TNF-α-producing DCs in the skin, however, the percentage was still significantly higher than that of the control treatment (Figure 5D). Following on from the increase in inflammatory DCs, effector T cells, such as Th1 and IL-17A+CD4+ T (Th17) cells, were both increased in the IMQ-treated ears, accompanied by abundant neutrophil accumulation. However, accumulation of these cells was significantly suppressed by LTA treatment (Figure 5E–G). 

Taken together, LTA suppresses TLR ligand-induced acute skin inflammation by suppressing the inflammatory responses of DCs in the skin.

## 3. Discussion

TLR signaling has an important role in immune cells to control the inflammatory response [14]. The response is not only for exogenous ligands, such as those produced from bacteria and fungus, but also for auto-antigens derived from their own tissue [15,16]. TLR signaling triggered in the recognition of a ligand leads to the activation of downstream factors, such as MyD88, TNF receptor associated factor (TRAF) and protein kinase B (PKB as well as Akt), and eventually, several transcription factors are driven to induce cytokine production and other inflammatory responses [17]. The signaling pathway has been well characterized after the identification of TLRs, and the causes of some of inflammatory diseases were revealed as TLR-dependent inflammation [18]. In DCs, TLR signaling is also an important factor to induce various immunological responses [19]. As one of the gatekeepers in the immune system, DCs frequently come into contact with many endogenous and exogenous simulators which can be antigens to activate T cells [20]. In this process, the TLRs are activated by several ligands so that the function is enhanced as a similar mechanism to immunization using an adjuvant in the DCs [21].

However, our immune system requires a suppressive function to prevent overactivation and autoreactivity. We hypothesized that an exogenous substance may be able to attenuate or regulate DC-based immune responses especially in a TLR-dependent manner. Therefore, we performed a BMDCs stimulation assay with various combinations of ligands, and eventually found that LTA suppressed the TLR-mediated cytokine production and cellular activation in the DCs (Figure 1, Figure 2 and Figure 3). LTA dramatically suppressed TNF-α production in the TLR ligand-stimulated BMDCs (Figure 1). The suppressive effect was obvious in LPS, PGN and IMQ stimulation because TNF-α production was dramatically suppressed by co-treatment of LTA, even though the dose was relatively low in the culture (Figure 1A,B,E). The suppressive signal of LTA was TLR2-dependent in the BMDCs, because the TLR2 blocking suppressed TNF-α production which was induced by LPS or IMQ stimulation (Figure 2A and Supplementary Figures S1 and S2). Our data on the determination of the responsive receptor for LTA are strongly supported by the evidence that LTA is dominantly recognized by TLR2 [22]. However, the possibility that LTA works through other receptors except TLRs must be considered. In fact, we previously reported that C-type lectin receptors (CLRs) may be bound and activated by LTA in the high dose treatment [23]. LTA treatment suppressed the downstream signaling pathway of TLR 4 in LPS-stimulated BMDCs (Figure 2B,C). We analyzed MyD88, pErk1/2, pp38 and pp65 as indispensable elements in the pathway of TLR4-related cytokine production, however, other factors, such as mitogen-activated protein kinase (MAPK) and c-Jun N-terminal kinase (JNK), also must be analyzed to complete the characterization of the LTA-based suppressive effect in DCs.

The suppressive effect of LTA in the immune response was also confirmed in antigen presentation to CD4+ T cells activation in BMDCs (Figure 3 and Figure 4). The BMDCs treated with LPS or IMQ upregulated the generation of Th1 cells in an antigen-specific manner. The responses were both strongly suppressed by co-treatment of LTA in the TLR ligand stimulation, so that the percentage of Th1 cells was significantly decreased in the culture (Figure 4, Supplementary Figure S4). The underlying mechanism might be based on the downregulation of MHC class II, CD80 and CD86 in the LTA-treated BMDCs (Figure 3, Supplementary Figure S4). This phenomenon was remarkably interesting and may be understood as a substantial mechanism in the regulation of adaptive immune response by LTA treatment.

To confirm the immunosuppressive effect of LTA in a practical inflammation model, we generated acute skin inflammation in mice eras by using IMQ as well as other TLR ligands (Figure 5, Supplementary Figure S7). The IMQ-induced skin inflammation was dramatically suppressed in the ears which received co-treatment of LTA. The ear thickness was consequently decreased in the mice (Figure 5A,B). We obtained the same results in other TLR ligand-induced skin inflammation models (Supplementary Figure S7). As a consistent phenomenon with an in vitro study, the inflammatory response, such as TNF-α production, in the skin-accumulated DCs was significantly suppressed by LTA treatment (Figure 5D). This is strong evidence that our initial finding of a LTA-originated suppressive effect in BMDCs can be adapted into an in vivo environment. While we must consider the possibility of a comprehensive immunological event in the LTA-mediated suppressive effect in the inflamed lesion, because Th1 and Th17 cells as well as neutrophils accumulation were completely parallel to the accumulation of DCs and inflammatory grade in the ears (Figure 5E–G). These data suggest that both adaptive and innate immune responses were regulated by LTA treatment in the TLR ligand-induced skin inflammation with DC-based manner. However, the suppression of DCs activity by LTA treatment should be an initial step in the immunosuppressive event in our hypothesis, because neither mice CD4+ T cells nor neutrophils were influenced directly in the LTA treatment (unpublished data).

These findings can be also adapted to the concept of symbiosis of commensal bacteria. *Staphylococcus aureus* (*S. aureus*) is one of the commensal bacteria and LTA is the major component in the bacterial cell wall [23]. LTA may suppress or regulate the host immune response, which is activated by several substances produced from the bacteria itself. As a consequence, the immunosuppressive effect of LTA may let the bacteria survive in the host without critical inflammation. A previous study showed that LTA has immunogenicity and induces an inflammatory response by the activation of immune cells [24], however, this concept is recently denied. The inflammatory responses observed in the study was originated from the contamination of lipopeptide in the LTA, therefore, a purified LTA did not show any inflammatory response in immune cells [25]. Our study used a pre-treated LTA with lipoprotein lipase, and the result also showed that LTA treatment did not induce cytokine production and cellular activation in the BMDCs (Figure 1F and Figure 3E and Supplementary Figures S1, S3 and S4).

To confirm the interesting immunosuppressive effect of LTA, further experiments must be performed using another DC model, such as primary DC. In vivo studies of other inflammatory diseases are necessary to reveal the effectiveness of LTA’s anti-inflammatory effect not only for TLR ligand-induced inflammation but also for that derived from other factors. In addition, we are interested in whether this anti-inflammatory response of LTA is conserved in other immune cells, especially myeloid lineages such as macrophages. Once all the mechanisms in the LTA-based anti-inflammatory response were revealed in immune cells, LTA may be used as a natural anti-inflammatory agent not only for daily care but also for clinical treatment. The bacterial ligand has potential in immune regulation. The study to identify an effective substance in bacterial components must be continued in both basic and clinical immunology.

## 4. Materials and Methods

### 4.1. Reagents and Antibodies

Lipopolysaccharide (LPS; *E. coli* origin), Pam3CSK4, peptidoglycan (PGN; *S. aureus* origin), Poly (I:C), imiquimod (IMQ) and lipoteichoic acid (LTA; *S. aureus* origin) were all purchased from Invivogen (San Diego, CA, USA). LTA was treated with lipoprotein lipase to inactivate contaminated lipoprotein before use in each experiment. Phorbol 12-myristate 13-acetate (PMA), ionomycin and ovalbumin (OVA) peptide (OVA_323–339_) were all purchased from Sigma Aldrich (St. Louis, MO, USA). In addition, 5% IMQ cram (Aldara) was purchased from 3M Health Care Limited (Loughborough, UK). Recombinant murine granulomacropahge colony stimulating factor (rmGM-CSF) was purchased from Peprotech (Rocky Hill, NJ, USA). Cytofix/Cytoperm kit with GoldiStop^TM^ was purchased from BD Bioscience (Franklin Lakes, NJ, USA). PerFix-p Kit was purchased from Beckman Coulter (Indianapolis, IN, USA); anti-TLR2 (QA16A01), anti-TLR3 (11F8), anti-TLR4 (MTS510), anti-CD80 (16-10A1), anti-CD86 (GL-1), anti-CD3 (17A2), anti-CD4 (GK1.5), anti-CD8a (53-6.7), CD69 (H1.2F3) and 7-amino-actinomycin D (7-AAD) were all purchased from BioLegend (San Diego, CA, USA). Anti-TLR1 (eBioTR23 (TR23)), Anti-TLR7 (4G6), anti-I-A/I-E (M5/114.15.2), anti-interferon gamma (IFN-γ) (XMG1.2), anti-CD16/CD32 (2.4G2) (93) were all purchased from Thermo Fisher Scientific (Waltham, MA, USA). Anti-MyD88 (4D6) was purchased from Novus Biologicals (Centennial, CO, USA). Anti-pErk (pT202/pY204) (20A), anti-p38 (pT180/pY182) (36/p38) and anti-NF-κB p65 (pS529) (K10-895.12.50) were purchased from BD Bioscience (Franklin Lakes, NJ, USA). The isotype-matched control for each antibody was purchased from the same company.

### 4.2. Mice

C57BL/6J and OT-II mice (B6.Cg-Tg (TcraTcrb) 425Cbn/J) were purchased from The Jackson Laboratory (Bar Harbor, ME, USA). All mice were maintained under specific pathogen-free (SPF) conditions with free access to water and food in 12 h day/night cycle. Gender-matched mice between 8 and 12 weeks of age were used for each experiment. All the animal experiments were carried out in accordance with the guidelines of the animal welfare committee and the ethics committee of the institutes (Northwest A&F University, Protocol No. 15-10-874 and Central South University, Protocol No. 201503302).

### 4.3. Acute Skin Inflammation Model

To establish IMQ-induced acute skin inflammation model, the mice ears were treated with 5% IMQ cream or control cream for 5 days. The mice received intradermal (ID) injection of LTA (100 ug) or saline daily during the experimental period. At day 5, the mice were sacrificed, and the ears were used for analysis. To establish acute skin inflammation model using other TLR ligands, the mice ears were treated with TLR ligand (LPS: 1 μg, PGN: 100 μg, Pam3CSK4: 1 μg or Poly (I:C): 100 μg) by intradermal (ID) injection. The mice ears received ID injection of LTA (100 μg) or vehicle control twice (in the meantime and post-24 h of TLR ligand treatment) during the experimental period. After 48 h, the mice were sacrificed, and the ears were used for analysis.

### 4.4. Mouse Primary Cell Isolation

Splenocytes were prepared from spleen by following a method described in a previous report [26]. Briefly, the extracted spleen was crushed on a 70 μm cell strainer with cell culture medium (RPMI 1640 supplemented with 10% fetal bovine serum (FBS), 100 U/mL penicillin and 100 mg/mL streptomycin). The cell suspension was filtered through a 70 μm cell strainer again and then washed with cell culture medium. After centrifugation at 300 g for 5 min, the cell pellet was resuspended in 1 × RBC lysis buffer at RT for 10 min. After being washed twice with the cell culture medium, the cells were precipitated by centrifugation at 300 g for 5 min. The cells were resuspended in cell culture medium and used as splenocytes. Skin leukocytes were isolated by following a method described in a previous report, with modifications [27]. Briefly, the extracted ear piece was washed with tissue washing buffer (RPMI 1640 supplemented with 10% fetal bovine serum (FBS), 10 mM 4-(2-hydroxyethyl)-1-piperazineethanesulfonic acid (HEPES) and 100 U/mL penicillin, 100 mg/mL streptomycin) at 37 °C for 30 min with gentle shaking. The ear piece was incubated at 37 °C for 30 min with dispase working solution (tissue washing buffer containing 0.25 mg/mL of dispase) to separate the epidermal and dermal sheets from cartilage. These sheets were cut with scissors into the smallest possible pieces (−1 mm) and then the skin fragments were incubated at 37 °C for 30 min in collagenase working solution (tissue washing buffer containing 1 mg/mL collagenase D and 0.01% DNase). The digested sample was filtered through a 70 μm cell strainer, then undigested skin pieces were crushed on the strainer. The strainer was washed with cell culture medium, then all flow through was passed through a 5 mL syringe with a 22 G needle to make single cell suspension. Mouse bone marrow leukocytes were obtained from the tibia and femur by following a method described in a previous report [28]. Briefly, the cells were flushed out from the tibia and femur by a 10 mL syringe with a 27 G needle containing cell culture medium. The cell suspension was filtered through a 70 μm cell strainer and washed once with cell culture medium, then the cells were treated with the 1 × RBC lysis buffer at RT for 10 min. After being washed twice with cell culture medium, the cells were used as bone marrow leukocytes. Splenic CD4+ T cells were isolated from OT-II mice-originated splenocytes by using MagniSort^TM^ Mouse CD4 T cell Enrichment Kit (Thermo Fisher Scientific, Waltham, MA, USA). The whole procedure for the cell isolation kit was performed by following the manual. The cell purity was checked by flow cytometry and the purified cell suspension with over 90% of CD4+ T cells was used for the experiment.

### 4.5. Mouse Bone Marrow Dendritic Cells (BMDCs) Preparation

Mouse BMDCs were prepared by following a method described in a previous report, with minor modifications [18]. At day 0, 2.0 × 10^6^ of bone marrow leukocytes were suspended in 10 mL of DC culture medium (cell culture medium containing 20 ng/mL of rmGM-CSF), and the cells were seeded on a 100 mm dish. At day 3, 10 mL of the fresh DC culture medium was added to the culture. At day 6 and 8, half of the cultured medium was collected and centrifuged, then the cell pellets were resuspended in 10 mL of fresh DC culture medium. The cell suspension was put back into the original plate. At day 10, cells were ready to use for each experiment. The differentiated BMDCs condition was checked by flow cytometry in every culture. The cell suspension with over 90% of CD11c+ and CD80low, CD86low and I-A/I-E+ in the CD11c+ population was used for experiment.

### 4.6. BMDCs Stimulation Assay

BMDCs (1.0 × 10^6^) were seeded on 12-well plate with cell culture medium. The cells were simulated with TLR ligand (LPS: 100 ng/mL, PGN: 10 ug/mL, Pam3CSK4: 100 ng/mL, Poly (I:C): 10 ug/mL or IMQ: 10 ug/mL) or vehicle control. Some cultures were further treated with LTA (0.1, 0.5, 1 and 5 ug/mL) or vehicle control at 37 °C for 24 h. After the stimulation, the cultured medium was harvested for cytokine ELISA and was kept at −80 °C until use. In some cultures, the treated cells were harvested for flow cytometry.

### 4.7. TLR2 Blocking in TLR Ligand-Stimulated BMDCs

BMDCs (1.0 × 10^6^) were seeded on 12-well plate with cell culture medium. The cells were pre-treated with anti-TLR2 mAb (10 ug/mL) or isotype antibody at 37 °C for 1 h. Then, the cells were treated with TLR ligand (LPS: 100 ng/mL or IMQ: 10 μg/mL) combined with LTA (5 μg/mL) or vehicle control at 37 °C for further 24 h. The cultured medium was harvested for cytokine ELISA and was kept at −80 °C until use. In some cultures, the treated cells were harvested for flow cytometry.

### 4.8. In Vitro Antigen Presentation and T Cell Activation

BMDCs (1.0 × 10^5^) were mixed with OT-II CD4+ T cells (5.0 × 10^5^) on 96-well plate in cell culture medium. OVA peptide (OVA_323–339_: 10 ug/mL) was added into cultures and then samples were treated with single or combination TLR ligands (LPS or IMQ LPS or IMQ+LTA, LPS). The ligand concentrations were LPS: 100 ng/mL, IMQ: 10 μg/mL and LTA: 5 ug/mL. Some cultures were treated with LTA only or vehicle control (no-Ag negative control). The culture was incubated at 37 °C for 72 h. At the last 5 h, the cells were re-stimulated with PMA (500 ng/mL) and ionomycin (1 ug/mL) in the presence of GolgiStop^TM^. The IFN-γ+CD4+ T (Th1) cells and CD69 expression in Th1 cell populations were analyzed by flow cytometry.

### 4.9. Flow Cytometry

Cell surface markers and intracellular proteins were analyzed by a flow cytometer (FACSCanto and LSR-II; BD Biosciences) with the fluorochrome-conjugated monoclonal antibodies described in reagents and antibodies. The cells were initially incubated with FcγRII/III blocker (anti-CD16/32; 2.4G2) at 4 °C for 10 min. For surface marker staining, the cells were incubated with the antibody at 4 °C for 30 min. Intracellular protein staining was performed by using a Cytofix/CytoPerm Kit or PerFix-p Kit by following the manual. Briefly, the cells stained with the antibody for the surface marker were fixed and permeabilized at 4 °C for 20 min. The cells were incubated with the antibody for staining of the target proteins at 4 °C for 30 min. The dead cells were excluded by forward scatter, side scatter and 7-AAD gating. All data were analyzed by BD FACS Diva (BD bioscience, Franklin Lakes, NJ, USA) or FlowJo (Tree Star; Ashland, OR, USA).

### 4.10. Cytokine Measurement by Enzyme-Linked Immuno Sorbent Assay (ELISA)

The cytokine (TNF-α) produced from the stimulated cell was measured by ELISA Ready-SET-Go!™ Mouse TNF-α Kit (Thermo Fisher Scientific, Waltham, MA, USA). The whole procedure was performed by following the manual.

### 4.11. Statistical Analyses

Student’s *t*-test was used to analyze the data for significant differences. Values of *p* < 0.05, *p* < 0.01 and *p* < 0.001 were regarded as significant.

## Supplementary Materials

The following are available online at https://www.mdpi.com/2076-0817/9/10/825/s1, Figure S1: The blocking efficiency of anti-TLR2 mAb in cytokine production of TLR ligand-stimulated BMDCs, Figure S2: The immunosuppressive effect of LTA is TLR2-dependent in IMQ-stimulated BMDCs, Figure S3: The blocking efficiency of anti-TLR2 mAb in signaling pathway of TLR ligand-stimulated BMDCs, Figure S4: The blocking efficiency of anti-TLR2 mAb in cellular activation of TLR ligand-stimulated BMDCs, Figure S5: The suppressive effect of LTA is abolished by TLR2 blocking in surface molecule expression of IMQ-stimulated BMDCs, Figure S6: LTA suppresses the upregulation of IMQ-induced antigen presentation and effector CD4+ T cell generation in DCs, Figure S7: TLR ligand-induced acute skin inflammation is attenuated by LTA co-treatment.
