# Supplementary material for: Bacterial Lipoteichoic Acid Attenuates Toll-Like Receptor Dependent Dendritic Cells Activation and Inflammatory Response
Source: Pathogens. 2020 Oct 8;9(10):825. doi: 10.3390/pathogens9100825 (PMC7600882; doi:10.3390/pathogens9100825)
Supplement: Supplementary file 1 [file pathogens-09-00825-s001.pdf]

**Supplemental materials**

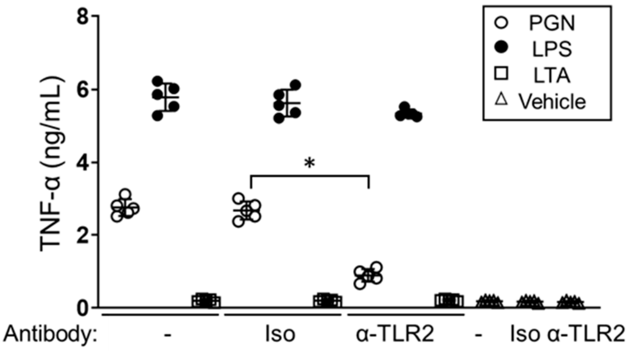


**Figure 1.** The blocking efficiency of anti-TLR2 mAb in cytokine production of TLR ligand stimulated BMDCs. BMDCs were pre-treated with anti-TLR2 mAb or isotype antibody at 37 °C for 1 h, then were stimulated with PGN (10 μg/mL), LPS (100 ng/mL), LTA (5 μg/mL) or vehicle control. The culture was further incubated at 37 °C for 24 h, then the TNF-*α* production was measured by ELISA. Data is shown as the mean ± SEM of five samples in independent experiments. The Student's *t*-test was used to analyze data for significant differences. Values of * $p < 0.001$ was regarded as significant.

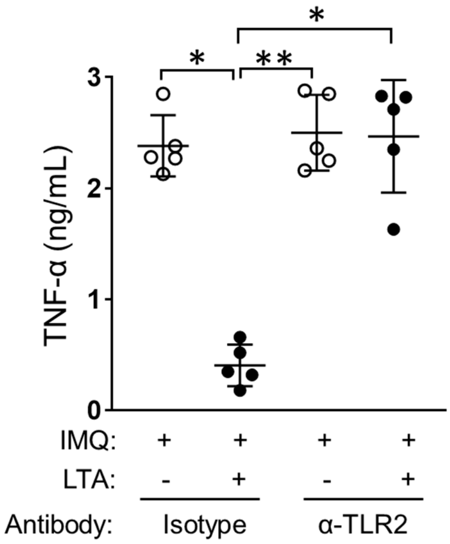


**Figure S2.** The immunosuppressive effect of LTA is TLR2 dependent manner in IMQ-stimulated BMDCs. BMDCs were pre-treated with anti-TLR2 mAb or isotype antibody at 37 °C for 1 h, then were stimulated with IMQ (10 μg/mL) combined with LTA (5 μg/mL) or vehicle control. The culture was further incubated at 37 °C for 24 h, then the TNF-*α* production was measured by ELISA. Data is shown as the mean ± SEM of five samples in independent experiments. The Student's *t*-test was used to analyze data for significant differences. Values of * $p < 0.01$ and ** $p < 0.001$ were regarded as significant.

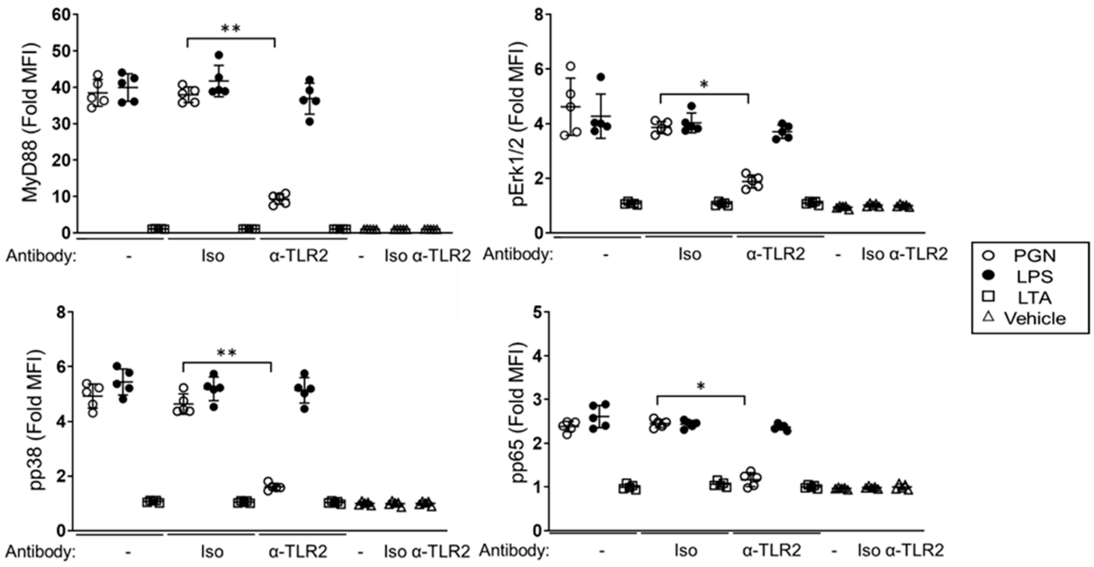


**Figure S3.** The blocking efficiency of anti-TLR2 mAb in signaling pathway of TLR ligand stimulated BMDCs. BMDCs were pre-treated with anti-TLR2 mAb or isotype antibody at 37 °C for 1 h, then were stimulated with PGN (10 μg/mL), LPS (100 ng/mL), LTA (5 μg/mL) or vehicle control. The culture was further incubated at 37 °C for 24 h, then, MyD88, pErk1/2, pp38 and pp65 expressions were analyzed by flow cytometry. Data is shown as the mean ± SEM of five samples in independent experiments. The Student's *t*-test was used to analyze data for significant differences. Values of * $p < 0.01$ and ** $p < 0.001$ were regarded as significant.

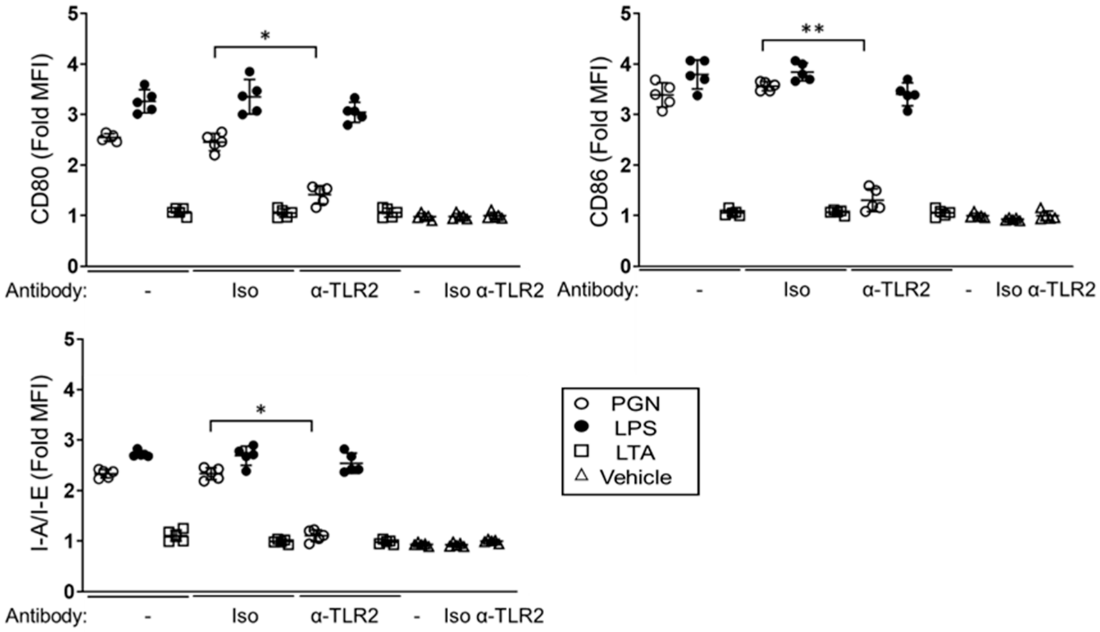


**Figure S4.** The blocking efficiency of anti-TLR2 mAb in cellular activation of TLR ligand stimulated BMDCs. BMDCs were pre-treated with anti-TLR2 mAb or isotype antibody at 37 °C

for 1 h, then were stimulated with PGN (10 μg/mL), LPS (100 ng/mL), LTA (5 μg/mL) or vehicle control (-). The culture was further incubated at 37 °C for 24 h, then CD80, CD86 and I-A/I-E expressions on the cell surface were analyzed by flow cytometry. Data is shown as the mean ± SEM of five samples in independent experiments. The Student's *t*-test was used to analyze data for significant differences. Values of * $p < 0.01$ and ** $p < 0.001$ were regarded as significant.

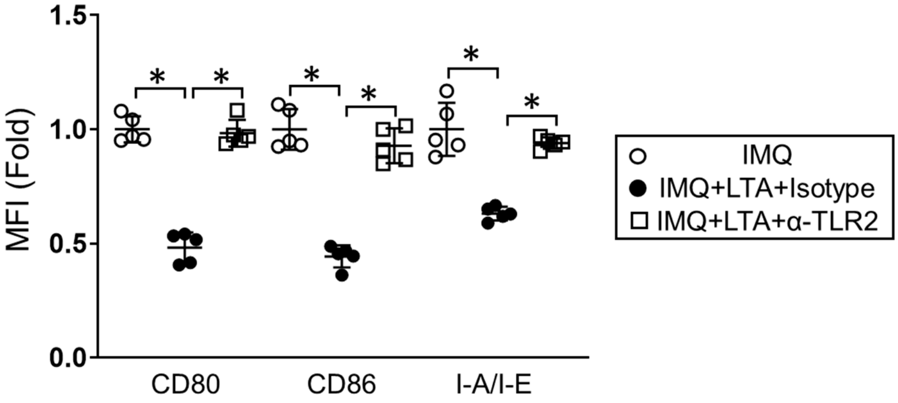


**Figure S5.** The suppressive effect of LTA is abolished by TLR2 blocking in surface molecule expression of IMQ-stimulated BMDCs. BMDCs were pre-treated with anti-TLR2 mAb or isotype antibody at 37 °C for 1 h, then stimulated with IMQ (10 μg/mL) combined with LTA (5 ug/mL) or vehicle control. The culture was further incubated at 37 °C for 24 h, then CD80, CD86 and I-A/I-E expressions on the cell surface were analyzed by flow cytometry. Data are shown as mean ± SEM of five samples in independent experiments. The Student's *t*-test was used to analyze data for significant differences. Values of * $p < 0.001$ was regarded as significant.

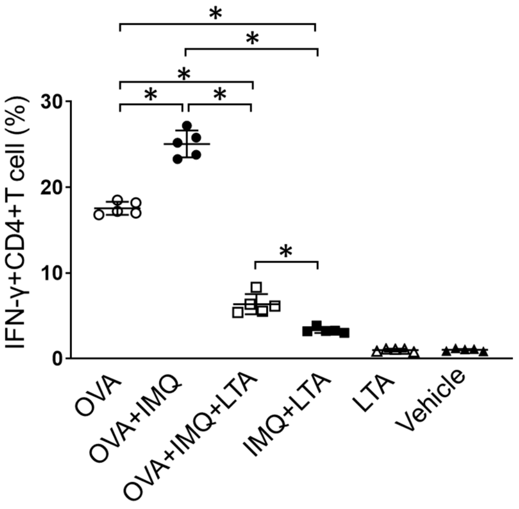


**Figure S6.** LTA suppresses the upregulation of IMQ-induced antigen presentation and effector CD4+ T cells generation in DCs. BMDCs were mixed with CD4+ T cells isolated from OT-II mice

with OVA peptide ($OVA_{323-339}$, 10 μg/mL). Some cultures were further treated with IMQ (10 μg/mL), LPS+LTA (5 μg/mL) or LTA. The samples treated with LTA-only or vehicle solution was negative control (no-Ag). The cultures were incubated at 37 °C for 72 h. At the last 5 h of culture, the cells were re-stimulated with PMA/ionomycin. The percentage of IFN-γ+CD4+ T (Th1) cells was analyzed by flow cytometry. Data are shown as the mean ± SEM of five samples in independent experiments. The Student's *t*-test was used to analyze data for significant differences. Values of * $p < 0.01$ was regarded as significant.

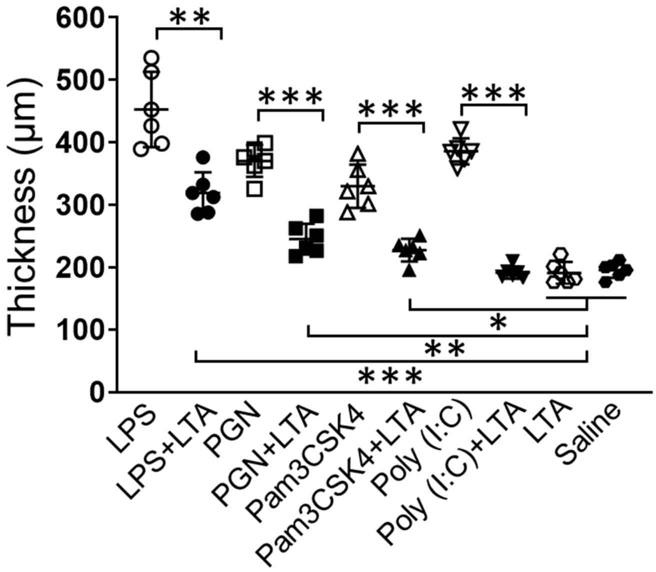


**Figure S7.** TLR ligand-induced acute skin inflammation is attenuated by LTA co-treatment. The TLR ligands (LPS: 1 μg, PGN: 100 μg, Pam3CSK4: 1 μg or Poly (I:C): 100 μg) was ID injected into the mice ears. The mice ears were also received ID injection of LTA (100 μg) or vehicle control for twice (at the meantime and post 24 h of TLR ligand injection) during experiment period. Some mice were received LTA-only or saline ID injection as control. After 48 h, the ear thickness was measured in H-E stained tissue section under microscope. Data are shown as the mean ± SEM of five samples in independent experiments. The Student's *t*-test was used to analyze data for significant differences. Values of * $p < 0.05$, ** $p < 0.01$ and *** $p < 0.001$ were regarded as significant.
